# A pan‐cancer blueprint of genomics alterations and transcriptional regulation of Siglecs, and implications in prognosis and immunotherapy responsiveness

**DOI:** 10.1002/ctm2.1262

**Published:** 2023-05-22

**Authors:** Song Mei, Yixuan Huang, Yan Zhao, Xinlei Zhang, Peng Zhang

**Affiliations:** ^1^ Beijing Key Laboratory for Genetics of Birth Defects Beijing Pediatric Research Institute MOE Key Laboratory of Major Diseases in Children; Rare Disease Center, Beijing Children's Hospital, Capital Medical University, National Center for Children's Health Beijing China; ^2^ Shanghai Institute of Immunology Shanghai Jiao Tong University School of Medicine Shanghai China; ^3^ Beijing ClouDNA Technology Co., Ltd. Beijing China; ^4^ Department of Otolaryngology Head and Neck Surgery, Beijing TongRen Hospital Capital Medical University Beijing China; ^5^ Division of Immunotherapy Institute of Human Virology University of Maryland School of Medicine Baltimore Maryland USA

Dear Editor,

Sialic acid‐binding immunoglobulin‐like lectins (Siglecs), which are aberrantly expressed on tumor cells, have become vital regulatory molecules in the development of tumor microenvironment (TME).^1^, ^2^ But so far, there are limited systematic studies about the genomic alterations, expression patterns and clinical significance of the Siglec genes across various cancer types.^3^, ^4^ To fill this gap, molecular alterations involving a total of 14 Siglec genes were examined across 10 967 human cancer samples representing 32 major types. And, we overlaid the expression signatures on single‐cell RNA‐sequencing (scRNA‐seq) datasets, enhancing spatial annotation of expression‐driven clusters in multiple cancers. Besides, correlation analyses revealed expression value of specific Siglec genes was associated with patient survival and can confer responsiveness to cancer immunotherapy.

As illustrated in Figure 1A, the four gene members of the Siglecs family including *CD169*, *CD22*, *MAG*, and *Siglec‐15* are highly homologous. They are also proven to be orthologues in all mammals analyzed by previous research.^5^ The remaining 11 Siglec genes are called *CD33*‐related Siglecs that were considered to evolve from duplication of the *CD33*. Siglecs are attracting interest as immune checkpoint targets for the creation of therapies that use them to enhance an anticancer immune response; hence, we here focus on the molecular landscape of genomic and transcriptomic data of the Siglec genes across 32 tumor types (as ‘pan‐cancer cohort’) in The Cancer Genome Atlas (TCGA) for further analysis. To identify genomic aberrations of the Siglecs genes at the pan‐cancer level, mutation and copy number variations (CNV) analyses were performed by using the cBioPortal tools and definitions.^6^ As shown in Figure 1B, the distribution plot shows that the Siglecs alteration frequencies and CNVs were relatively low, with seven cancer types harboring more than 20 percent of genomic alterations and 15 cancer types contained more than 10 percent of genomic alterations in at least one of the Siglec genes. Specifically, for somatic mutation profiles, there were somatic gene alterations of the Siglec genes in more than 20% of samples in five cancer types (Figure 2A). Skin cutaneous melanoma (SKCM, 47%), Lung squamous cell carcinoma (LUSC, 27%), Uterine corpus endometrial carcinoma (UCEC, 24%), Lung adenocarcinoma (LUAD, 23%) and Stomach adenocarcinoma (STAD, 21%) had the highest percentages of somatic mutations while thyroid carcinoma (0.6%) and Uveal melanoma (UVM, 1.3%) had the lowest. Besides, the type of somatic mutation and mutation site varied widely across all the Siglec genes, and the hot‐spot mutation site was not observed (Figure 2B). Furthermore, the Oncoprint representation (Figure 2C) revealed the tumors rarely had more than one mutationally altered gene within a category. The *SIGLEC1* (6%), *CD22* (4%) and *SIGLEC10* (4%) were the most frequently altered genes, while *SIGLEC15* and *SIGLEC16* had mutation frequencies < 0.5%. Although the frequency and type of copy number variation varied widely, it was noted that the Siglec genes had widespread recurrent chromosomal amplifications (Figure 2D). Among all cancer types, uterine cancer and ovarian cancer (OV) had the highest amplification frequency (>10%), which could be due to genomic instability. Esophageal carcinoma (ESCA) had the highest deletion frequency, marked by losses in the *SIGLEC15* (Figure 2E). *CD22* and *MAG* are the two most prominent copy number amplification genes in multiple cancer types, while *SIGLEC1*5 is almost the only one frequent deletion gene (Figure 2E).

**FIGURE 1:**
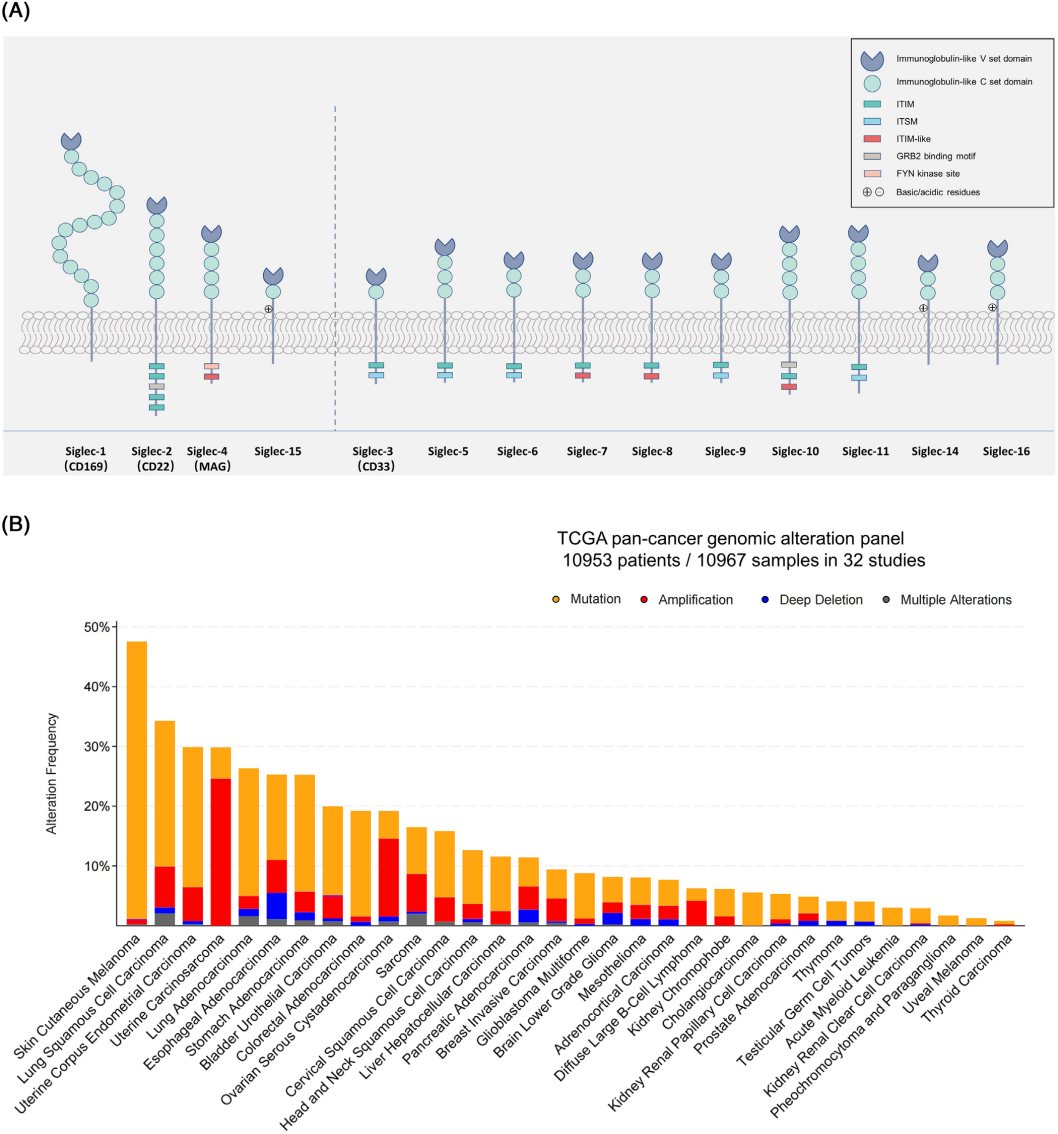
Siglecs family and its genomic alterations in cancers. (A) Siglec‐family proteins in humans. Siglecs are type 1 membrane proteins containing an amino‐terminal V‐set immunoglobulin domain that mediates sialic‐acid recognition and varying numbers of C2‐set immunoglobulin domains. Siglecs can be divided into two groups based on sequence similarity and evolutionary conservation. (B) The landscape of genomic aberrations in the Siglecs family genes in cancer. The frequency of alterations in Siglec genes across a pan‐cancer level with alteration rates for each mutation type are displayed in the left and top labels.

**FIGURE 2:**
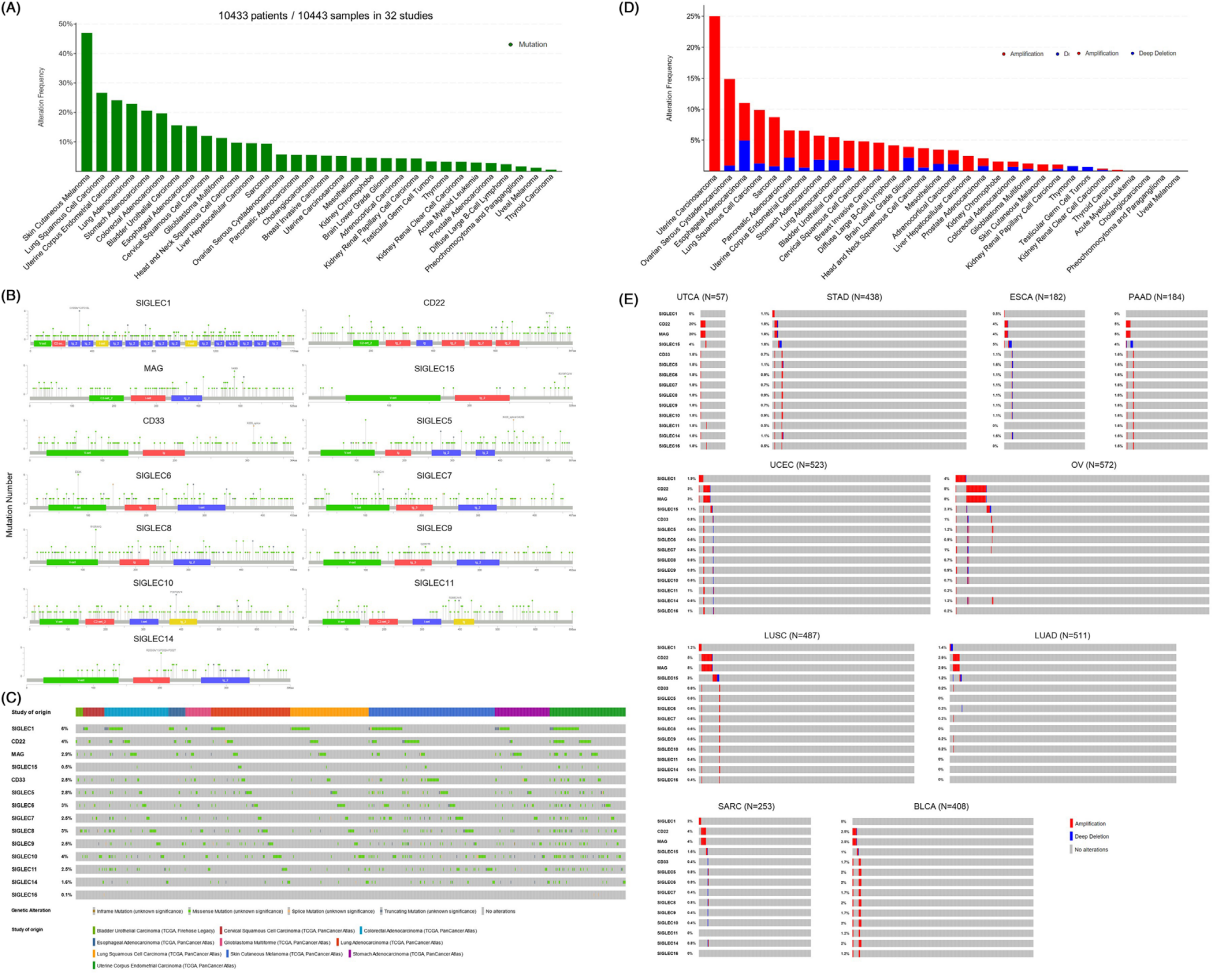
Genomics alteration landscape of Siglecs among different cancer types. (A) Mutational landscape. Distribution of total non‐silent mutation count among different cancer subtypes for Siglec genes. (B) Recurrent hotspot sites.Hot‐spot location of non‐silent mutations for the Siglec gene family. Mutations that result in substitutions are indicated. (C) Oncoprint mutation profile. The oncoprint plot for non‐silent mutations in each Siglec gene across all cancer types as identified in the TCGA PanCancer cohort. (D) Copy number variations landscape. Distribution of copy number variations (amplification and deletion) among different cancer subtypes for Siglec genes. (E) Oncoprint profile of copy number variations. The oncoprint plot for amplifications and deletions in each Siglec gene across all cancer types as identified in the TCGA pan‐cancer cohort.

To explore gene expression variation of the Siglec genes at a pan‐cancer level, we downloaded and analyzed the mRNA expression value derived from the RNA‐seq platform of all the Siglec genes. The relative expression level of the Siglecs family in tumor samples compared with normal tissue varied in different cancer types, and it is to be noted that all the Siglec genes expressed higher in metastasis samples compared with primary tumors (Figure S1). Unsupervised hierarchical clustering analysis identified specific patterns based on gene expression profile (Figure 3A). Specifically, the 14 genes produced distinct clusters that were dominated by cancer type. The *MAG* gene has a distinct expression value that was selectively highly expressed in glioblastoma multiforme (GBM), SKCM, and OV. The genes of *SIGLEC11*, *SIGLEC16*, *SIGLEC5*, *SIGLEC6*, *SIGLEC15*, *CD33* and *SIGLEC7* have a universally low expression value among all the cancer types, while *CD33* and *SIGLEC7* are selectively highly expressed in GBM and Sarcoma (SARC), and the *SIGLEC15* selectively highly expressed in SARC, UCEC and Bladder urothelial carcinoma (BLCA). *SIGLEC9*, *SIGLEC14* and *SIGLEC8* have a universally high expression value at a pan‐cancer level when compared with all other Siglec genes, but *MAG* was selectively highly expressed in GBM only. *SIGLEC1* and *CD22* were both high expressions on SKCM and OV, while *CD22* has a higher expression value in SARC and GBM. Next, we investigated the co‐expression value of all the Siglec genes in a pan‐cancer census, and a total of five clusters (including C1, C2, C3, C4 and C5) emerged when we grouped co‐expression patterns by cancer types (Figure 3B). Cluster C1 was enriched with STAD, SKCM and BLCA, while Cluster C2 was enriched with UCEC, OV and SARC. Cluster C3 was enriched with gastrointestinal tumors (including pancreatic adenocarcinoma [PAAD], ESCA, colon adenocarcinoma [COAD], and rectum adenocarcinoma [READ]) and cervical squamous cell carcinoma and endocervical adenocarcinoma and was characterized by more quantity of co‐expression pairs coupled with a lower correlation coefficient among the Siglec genes. The GBM is the only cancer type for Cluster C4 that has a distinct co‐expression pattern of the Siglec genes, which is consistent with the aforementioned gene expression pattern. The two lung cancer sub‐types, including LUAD and LUSC, constitute Cluster C5, which has a very similar co‐expression pattern of the Siglecs genes. To investigate the connection between genomic alterations and transcriptomic changes, we compared the expression level of Siglec genes in patients with various gene mutations and CNVs, respectively. The expression of several Siglec genes altered when missense mutation occurs, including *CD33*, *SIGLEC8* and *SIGLEC6*, while more transcriptomic changes were associated with CNV (Figure S2).

**FIGURE 3:**
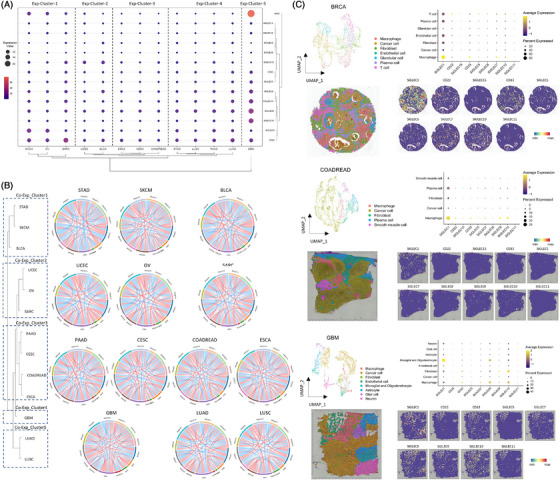
Expression patterns of Siglec genes at a pan‐cancer level. (A) The Siglecs gene expression signature at a pan‐cancer level. Color reflects the median expression of the Siglecs family across various cancer types. (B) Circos plot of mRNA co‐expression patterns at a pan‐cancer level. (C) The Uniform manifold approximation and projection (UMAP) plot shows the distinct clusters of spots in BRCA, COAD/READ and GBM (upper left). Dot plot of mean expression of the Siglecs gene for each spot cluster (upper right). The spot clusters are overlaid upon the histologic section (lower left). The spatial distribution of mean expression of Siglec genes upon the histologic section (lower right).

To clarify the mRNA expression of the Siglec genes in different cell types and further explore the spatial distribution characteristics of the Siglecs at a pan‐cancer level, we obtained both single‐cell RNA sequencing data and spatial transcriptomics of BRCA, COAD/READ, and GBM for analysis. The spots in spatial transcriptomics were clustered and named according to the enriched cell types in the spots. In BRCA, *SIGLEC1*, *CD33* and *SIGLEC7* were highly expressed in the macrophage‐enriched region, while *CD22*, *SIGLEC5* and *SIGLEC6* were highly expressed in T cell‐enriched region, and *SIGLEC10* was highly expressed in both regions (Figure 3C). In COAD/READ, all detected Siglecs showed higher expression in the Macrophage‐enriched region (Figure 3C). In GBM, the microglial and oligodendrocyte‐enriched region had high expression of *SIGLEC1*, *SIGLEC8* and *SIGLEC11*, while the macrophage‐enriched region exhibited high expression of *CD22*, *CD33* and *SIGLEC7* (Figure 3C). To validate these findings, we employed a deconvolution algorithm, xCell,^7^ to estimate the cell proportion of BRCA, COAD/READ and GBM samples in TCGA and analyzed the correlations between each cell group and Siglec gene. The results illustrated the expression level of Siglec genes were highly correlated with the proportion of Macrophage, especially M1 macrophage (Figure S3). These results indicated that Siglec genes widely existed in macrophages of the TME across various cancer types and had a higher expression in the microglial cells of GBM, which suggested that Siglecs may influence tumor progression and participate in the reconstruction of the TME by the regulation of tumor immunity and polarization of tumor‐associated macrophages. However, Siglec genes did not drive the interactions between immune cells and other cells (Figure S4).

Next, we used the large patient cohort available based on a 5‐year overall survival time to determine survival correlates in pan‐cancer studies. Many Siglec genes members were significantly associated with clinical outcomes (Figure 4A). Specifically, gene expression is significantly associated with worse patient outcomes, independent of cancer type, including *SIGLEC5*, *MAG* and *SIGLEC1*5. But, for some specific cancer types (LUAD and SKCM), *CD33*, *SIGLEC8* and *SIGLEC11* could serve as favorable prognosis markers for patients’ survival. Next, we employed a bioinformatics platform called tumor immune dysfunction and exclusion (TIDE)^8^ to query 14 Siglec genes to search the potential therapeutic targets in synergy with immune checkpoint blockade (ICB). Many Siglec genes are ranked by the Gene Prioritization module of TIDE to render the TME resistant to ICB (Figure 4B). These results prioritize many Siglecs that have the potential for developing novel immunotherapy targets. Given the link between Siglecs gene expression and TME, we further investigated whether the Siglecs expression was associated with immunotherapy responsiveness based on many published patient cohorts treated with ICB. As shown in Figure 4C, the expression level of *CD33*, *SIGLEC11* and *SIGLEC16* increased in melanoma patients responded to the combination therapy of anti‐PD1 (Pembrolizumab or Nivolumab) and anti‐CTLA4 (Ipilimumab) monoclonal antibody. *SIGLEC1*, *CD22*, *SIGLEC10* and *SIGLEC16* were also higher in Pembrolizumab or Nivolumab responsive melanoma patients. Moreover, *SIGLEC16* is associated with the response to Pembrolizumab in a gastric tumor cohort. These results indicate the treatment‐naive patients with higher expression of some Siglec genes (such as *SIGLEC16*) tend to be more sensitive to ICB.

**FIGURE 4:**
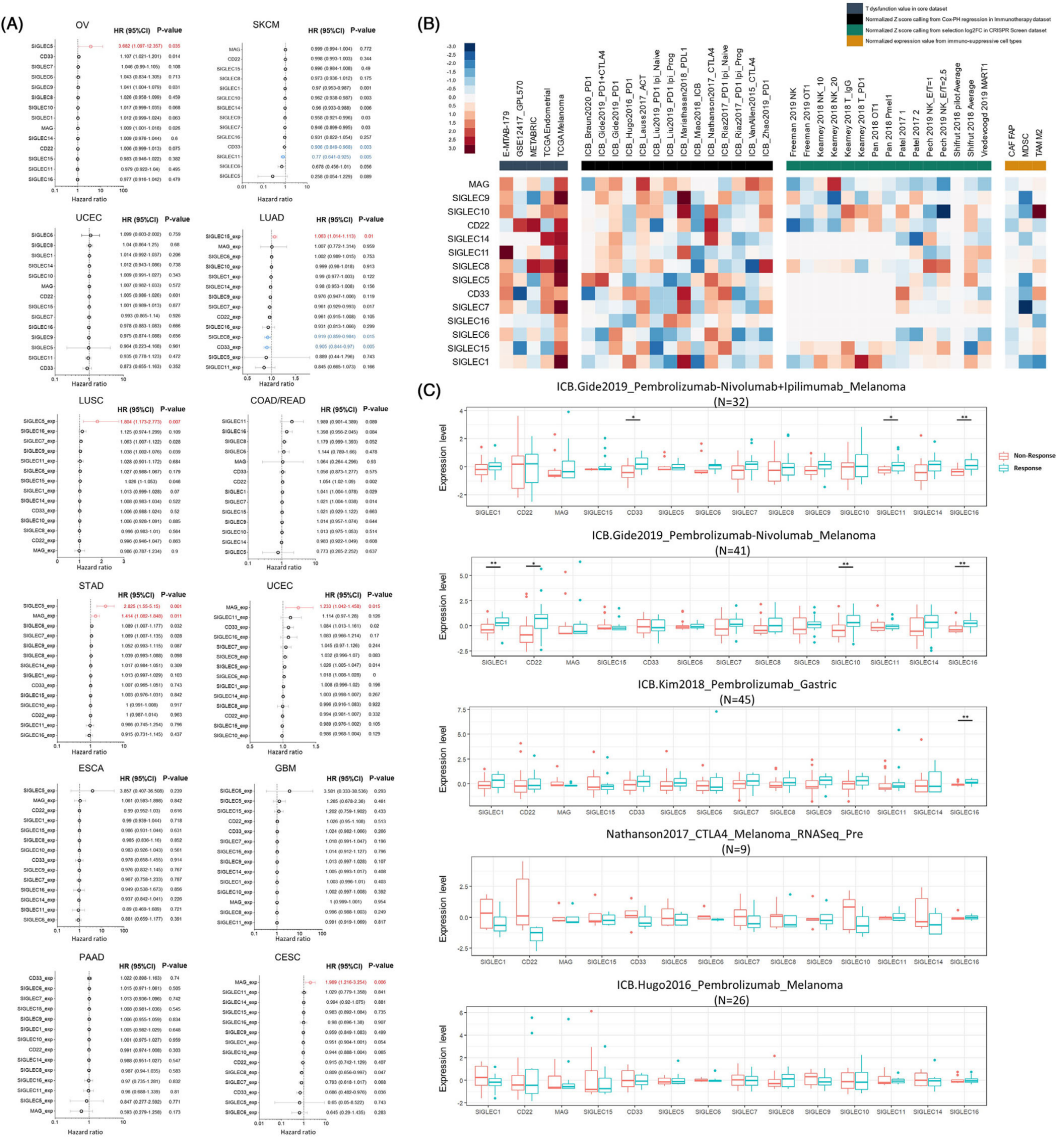
Effect of Siglecs expression on overall survival and anti‐cancer immune response. (A) Forest plots of hazard ratios by tumor type (with 95% confidence intervals) for 5‐year overall survival based on the Siglecs gene expression. Hazard ratios are based on gene expression level and a hazard ratio of more than 1 denotes the trend of expression upregulation with a worse outcome. The *p*‐value for overall survival correlation by meta‐analysis fixed effects model. (B) Prioritization of the Siglec genes with tumor immunity and immunotherapy response. Genes (row) are ranked by their weighted average value across four immunosuppressive indices (columns), including T cell dysfunction score, T cell exclusion score, association with ICB survival outcome, and log‐fold change (logFC) in CRISPR screens. **(C)** Distribution of the Siglecs gene family expression based on the public immunotherapy patient cohorts. Each data point represents one tumor sample of each patient cohort, and the *p*‐value was determined by the Wilcoxon test.

In summary, Siglecs signaling has exhibited an important function in anti‐tumor immunity in many previous studies.^9^ It is essential to explore their variation in genomic and transcriptomic levels. In addition to known mutation and copy number variations, our results dissected the dysregulated transcriptional level of each Siglec gene at both tissue and single‐cell levels having a distinct activation pattern, and a substantial fraction of cancers showed high activity without an associated canonical genomic alteration, suggesting various activation identity for Siglecs family. We believe our study provides a strong rationale for the development of pharmacologic inhibitors of Siglecs intending to augment immunotherapy.

## CONFLICT OF INTEREST STATEMENT

Y.H. and X.Z. are employees of the Beijing ClouDNA Technology Co. Ltd.; other authors declare no competing financial interests.

## FUNDING INFORMATION

Beijing Nova Program, Grant Number: Z211100002121044

## Supporting information

Supporting Information

Supporting Information

Supporting Information

Supporting Information

Supporting Information

## References

[1] Smith BAH , Bertozzi CR . The clinical impact of glycobiology: targeting selectins, Siglecs and mammalian glycans. Nat Rev Drug Discov. 2021;20:217‐243. doi:10.1038/s41573-020-00093-1 33462432 PMC7812346

[2] van de Wall S , Santegoets KCM , van Houtum EJH , et al. Sialoglycans and Siglecs can shape the tumor immune microenvironment. Trends Immunol. 2020;41:274‐285. doi:10.1016/j.it.2020.02.001 32139317

[3] Jiang K‐Y , Qi L‐L , Kang F‐B , Wang L . The intriguing roles of Siglec family members in the tumor microenvironment. Biomark Res. 2022;10:22. doi:10.1186/s40364-022-00369-1 35418152 PMC9008986

[4] Duan S , Paulson JC . Siglecs as immune cell checkpoints in disease. Annu Rev Immunol. 2020;38:365‐395. doi:10.1146/annurev-immunol-102419-035900 31986070

[5] Crocker PR , Paulson JC , Varki A . Siglecs and their roles in the immune system. Nat Rev Immunol. 2007;7:255‐266. doi:10.1038/nri2056 17380156

[6] Gao J , Aksoy BA , Dogrusoz U , et al. Integrative analysis of complex cancer genomics and clinical profiles using the cBioPortal. Sci Signal. 2013;6:pl1. doi:10.1126/scisignal.2004088 23550210 PMC4160307

[7] Aran D , Hu Z , Butte AJ . xCell: digitally portraying the tissue cellular heterogeneity landscape. Genome Biol. 2017;18:220. doi:10.1186/s13059-017-1349-1 29141660 PMC5688663

[8] Fu J , Li K , Zhang W , et al. Large‐scale public data reuse to model immunotherapy response and resistance. Genome Med. 2020;12:21. doi:10.1186/s13073-020-0721-z 32102694 PMC7045518

[9] van Houtum EJH , Büll C , Cornelissen LAM , Adema GJ . Siglec signaling in the tumor microenvironment. Front Immunol. 2021;12:790317. doi:10.3389/fimmu.2021.790317 34966391 PMC8710542

