# Supplementary material for: A pan‐cancer blueprint of genomics alterations and transcriptional regulation of Siglecs, and implications in prognosis and immunotherapy responsiveness
Source: Clin Transl Med. 2023 May 22;13(5):e1262. doi: 10.1002/ctm2.1262 (PMC10203536; doi:10.1002/ctm2.1262)
Supplement: Supplementary file 1 — Supporting Information [file CTM2-13-e1262-s003.docx]

**Supplementary Materials and Methods**

**Clinical Samples and Database**

We collected the largest publicly available cancer genomics database namely The Cancer Genome Atlas with genomic, transcriptomic, and clinical data. These datasets here are based on data generated by TCGA Research Network (http://cancergenome.nih.gov/). Molecular data from 11219 human cancers were aggregated from public repositories (https://portal.gdc.cancer.gov/). Tumors spanned 32 different TCGA projects, each project representing a specific cancer type, listed as follows: LAML, Acute Myeloid Leukemia; ACC, Adrenocortical carcinoma; BLCA, Bladder Urothelial Carcinoma; LGG, Brain Lower Grade Glioma; BRCA, Breast invasive carcinoma; CESC, Cervical squamous cell carcinoma, and endocervical adenocarcinoma; CHOL, Cholangiocarcinoma; COADREAD, Colorectal adenocarcinoma (combining COAD and READ projects); ESCA, Esophageal carcinoma; GBM, Glioblastoma multiforme; HNSC, Head and Neck squamous cell carcinoma; KICH, Kidney Chromophobe; KIRC, Kidney renal clear cell carcinoma; KIRP, Kidney renal papillary cell carcinoma; LIHC, Liver hepatocellular carcinoma; LUAD, Lung adenocarcinoma; LUSC, Lung squamous cell carcinoma; DLBC, Lymphoid Neoplasm Diffuse Large B-cell Lymphoma; MESO, Mesothelioma; OV, Ovarian serous cystadenocarcinoma; PAAD, Pancreatic adenocarcinoma; PCPG, Pheochromocytoma, and Paraganglioma; PRAD, Prostate adenocarcinoma; SARC, Sarcoma; SKCM, Skin Cutaneous Melanoma; STAD, Stomach adenocarcinoma; TGCT, Testicular Germ Cell Tumors; THYM, Thymoma; THCA, Thyroid carcinoma; UCS, Uterine Carcinosarcoma; UCEC, Uterine Corpus Endometrial Carcinoma. The immunological data files and annotated signature files of cancer samples were downloaded from NIH Genomic Data Commons (https://gdc.cancer.gov/about-data/publications/panimmune, February 2019). And we used the tumor immune estimation resource (TIMER) framework to comprehensively estimated the cell abundance of infiltrated immune cells in the tumor microenvironment [1].

**Single-cell sequencing and spatial transcriptomics data analysis**

The gene expression counts profile and H&E Staining images of BRCA (https://www.10xgenomics.com/cn/resources/datasets/human-breast-cancer-whole-transcriptome-analysis-1-standard-1-2-0), COADREAD (https://www.10xgenomics.com/cn/resources/datasets/human-colorectal-cancer-whole-transcriptome-analysis-1-standard-1-2-0), and GBM (https://www.10xgenomics.com/cn/resources/datasets/human-glioblastoma-whole-transcriptome-analysis-1-standard-1-2-0) were accessed from the 10X Genomics website (https://www.10xgenomics.com/cn/resources). For single-cell sequencing data studies, analyses were performed using R software (https://www.r-project.org/, version 4.1) and primarily using the package “Seurat”. To process data, cells were filtered, keeping only those cells with the number of genes detected per cell > 300 and < 4000, and percent mitochondrial genes < 0.10. Samples were then log-normalized and scaled whereby the number and two variables (unique molecular identifiers (UMIs) and percent mitochondrial genes) were regressed out. Clusters were determined using the first 15 principal components and graphed using UMAP dimensional reduction for each sample. Each cluster was defined based on clustering and marker genes. For spatial transcriptomics data studies, analyses were also performed using R software and primarily using the package “Seurat”. The function of “SCTransform” was used to normalize and scale the expression values, then clusters were determined using the first 30 principal components and graphed using UMAP dimensional reduction. Each cluster was defined based on clustering and cell-specific marker genes.

**Gene-set enrichment analysis**

Gene-set enrichment analysis was performed with the GSEA [2] program (version.3.0). The Broad Molecular Signatures Database (MSigDB v6.0) set H (hallmark gene sets) was used, which summarize and represent specific well-defined biological states or processes. The GSEA program was run with 1,000 permutations for statistical significance estimation, and the default signal-to-noise metric between the two phenotypes was used to rank all genes.

**xCell Analysis**

We use the xCell package (version 1.1.0) in the R environment to analyze the proportion of 64 immune and stroma cell types from gene expression data [3]. Gene expression matrices with row names as symbols and columns as samples were uploaded directly to calculate cell proportion matrices. Then Pearson correlation analysis between the cell proportion and the expression level of Siglec genes was conducted using corr.test function in the psych package (version 2.3.3).

**Cell–cell interaction network analysis**

Potential interactions between two cell clusters were predicted using the CellPhoneDB method (version 2.1.2, www.cellphonedb.org) based on the expression levels of receptor-ligand pairs through 1000 permutation tests [4]. The resulting adjacency matrices for all cell-cell interactions were displayed as dot-plots. Only gene pairs for receptor-ligand interactions in cell types of relevance were observed, and cell-cell interactions within identical biological lineages were omitted.

**Biostatistical Analysis**

Data were analyzed using an unpaired t-test with Welch's correction to compare between two groups. Fisher's exact test was used for enrichment analysis. For the differential expression analysis, the Mann-Whitney test with multiple testing adjustments (False Discovery Rate, FDR) determined the significant difference. The prognostic value of discrete variables was estimated by Kaplan–Meier survival curves, and the log-rank test was employed to estimate the significance among different survival curves. Statistical calculations were performed using GraphPad Prism software (GraphPad Software, San Diego, California) or R software (https://www.r-project.org/).

**References**

1. Li T, Fan J, Wang B, et al (2017) TIMER: A Web Server for Comprehensive Analysis of Tumor-Infiltrating Immune Cells. Cancer Research 77:e108--e110. https://doi.org/10.1158/0008-5472.CAN-17-0307

2. Subramanian A, Tamayo P, Mootha VK, et al (2005) Gene set enrichment analysis: A knowledge-based approach for interpreting genome-wide expression profiles. Proceedings of the National Academy of Sciences 102:15545–15550. https://doi.org/10.1073/pnas.0506580102

3. Aran D, Hu Z, Butte AJ (2017) xCell: digitally portraying the tissue cellular heterogeneity landscape. Genome Biol 18:220. https://doi.org/10.1186/s13059-017-1349-1

4. Efremova M, Vento-Tormo M, Teichmann SA, Vento-Tormo R (2020) CellPhoneDB: inferring cell-cell communication from combined expression of multi-subunit ligand-receptor complexes. Nat Protoc 15:1484–1506. https://doi.org/10.1038/s41596-020-0292-x
