# Supplementary figures and images for: A pan‐cancer blueprint of genomics alterations and transcriptional regulation of Siglecs, and implications in prognosis and immunotherapy responsiveness
Source: Clin Transl Med. 2023 May 22;13(5):e1262. doi: 10.1002/ctm2.1262 (PMC10203536; doi:10.1002/ctm2.1262)

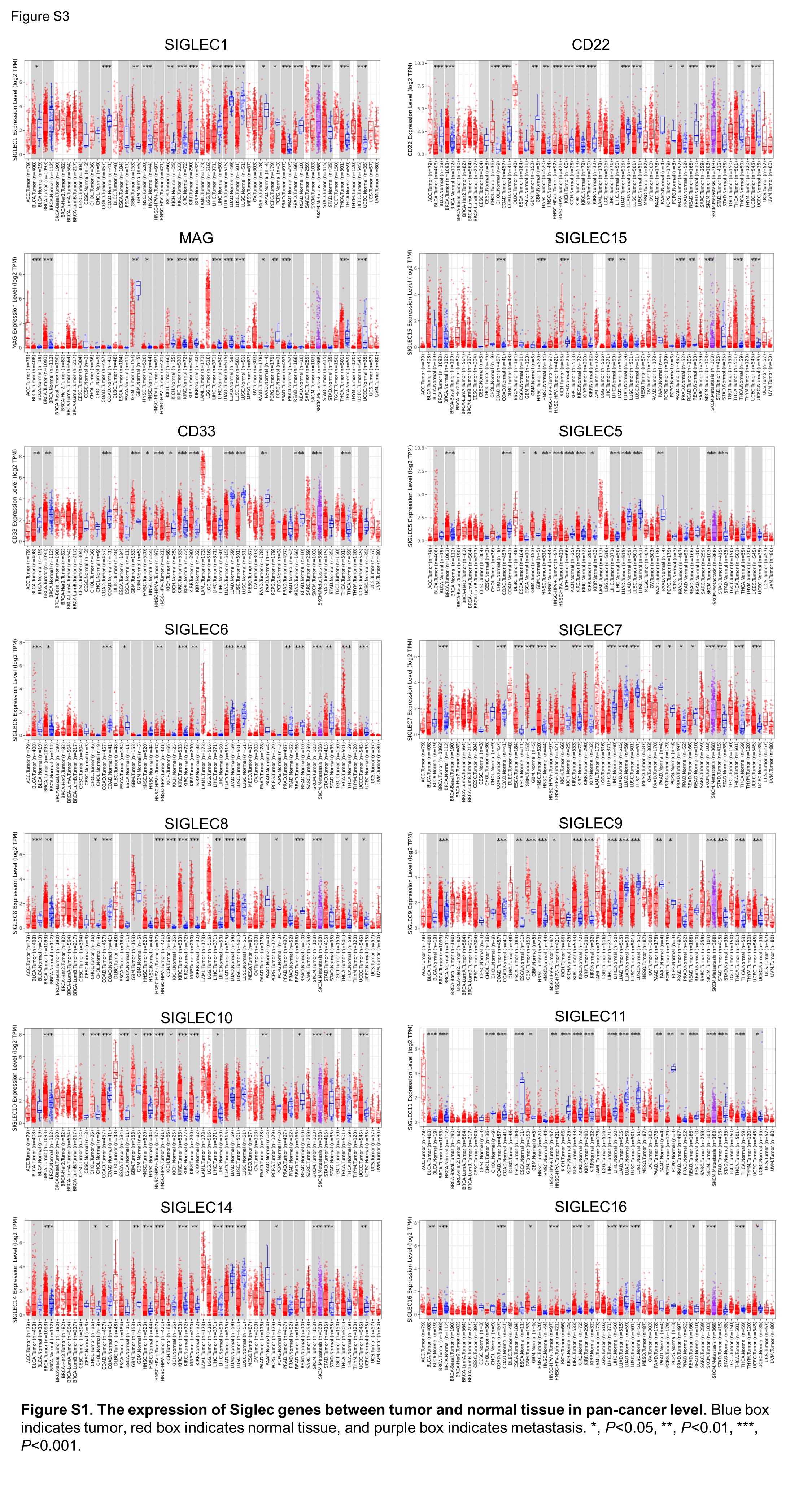

Supplement: Supplementary file 2 — Supporting Information [file CTM2-13-e1262-s002.jpg]

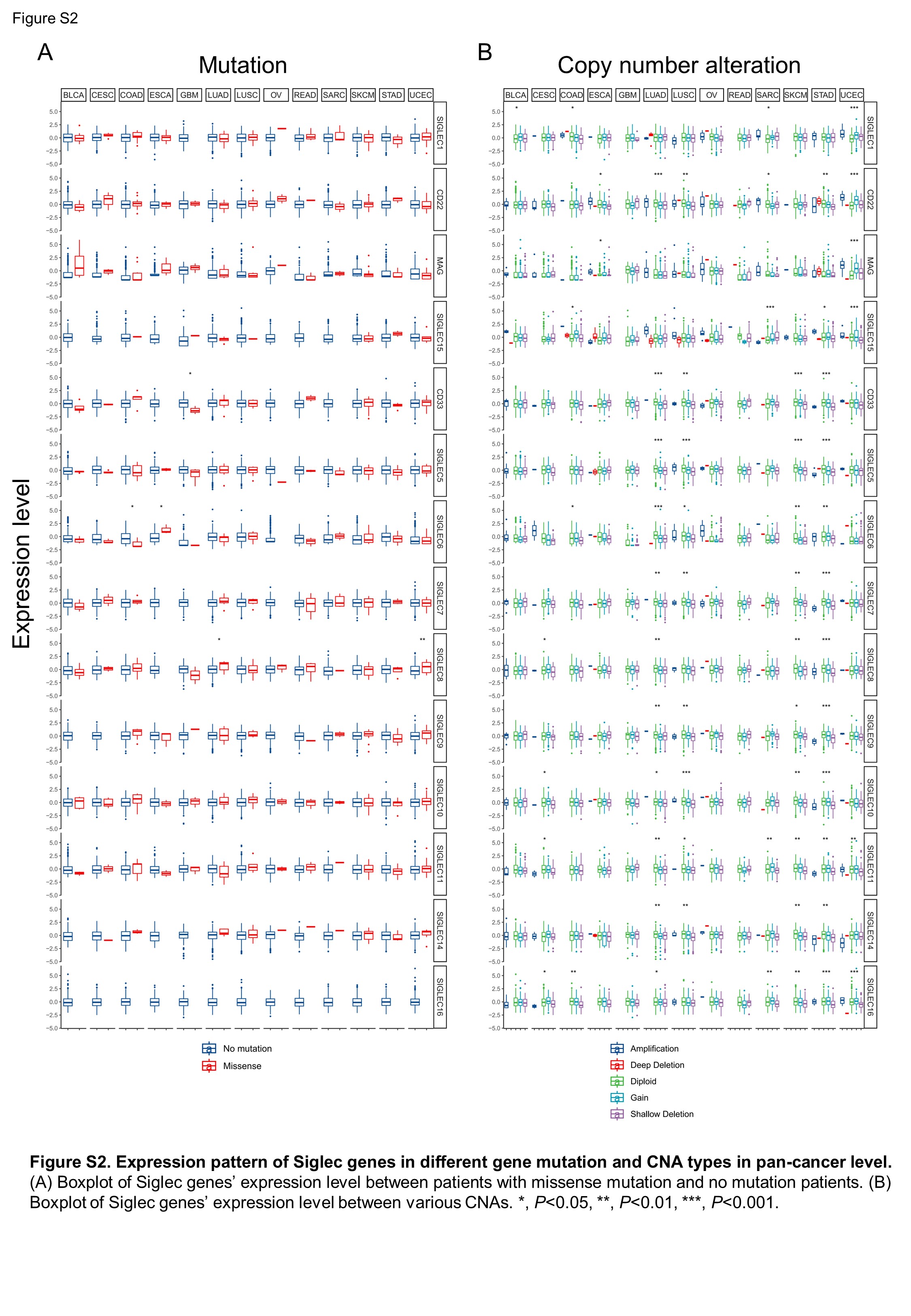

Supplement: Supplementary file 3 — Supporting Information [file CTM2-13-e1262-s001.jpg]

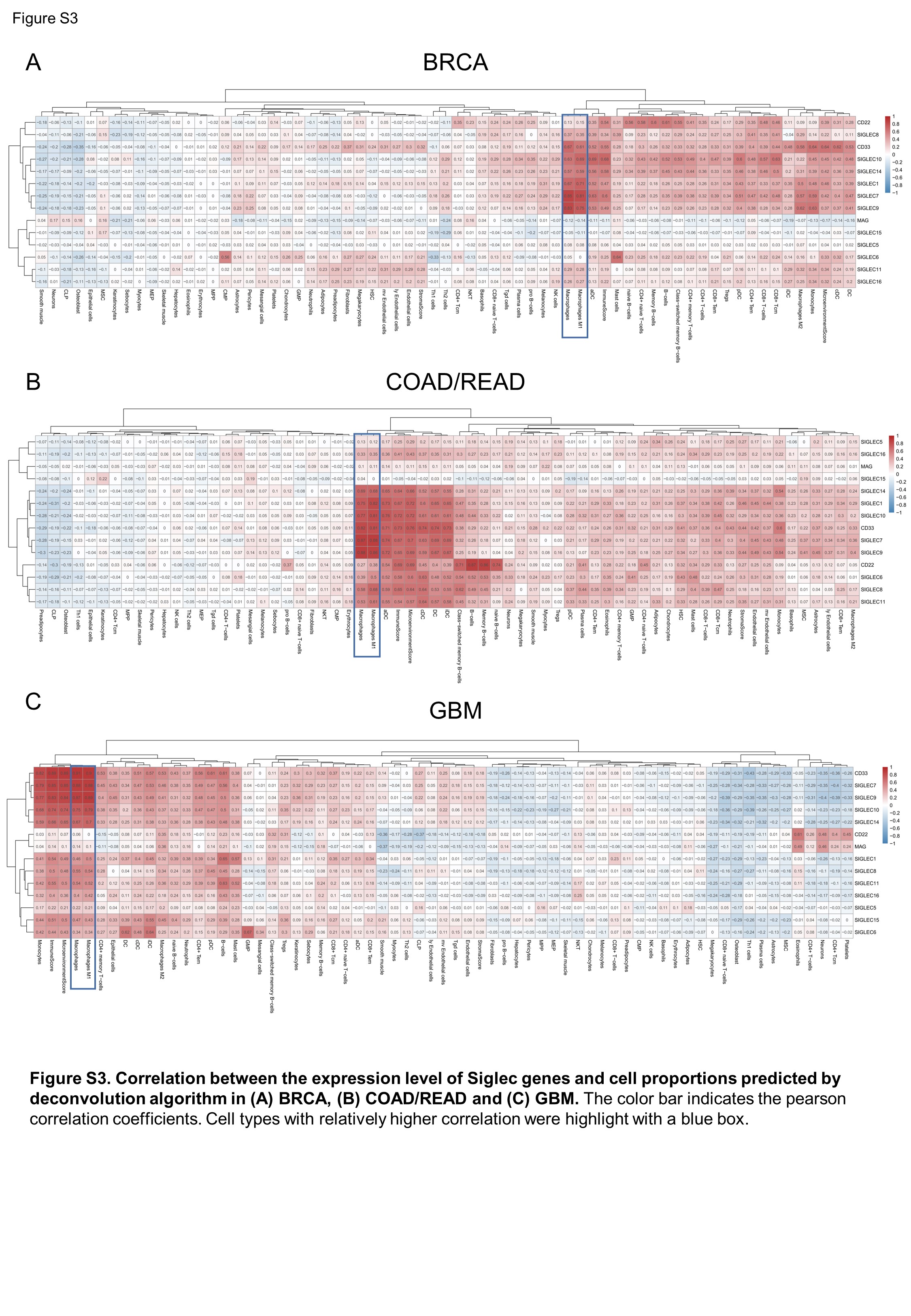

Supplement: Supplementary file 4 — Supporting Information [file CTM2-13-e1262-s005.jpg]

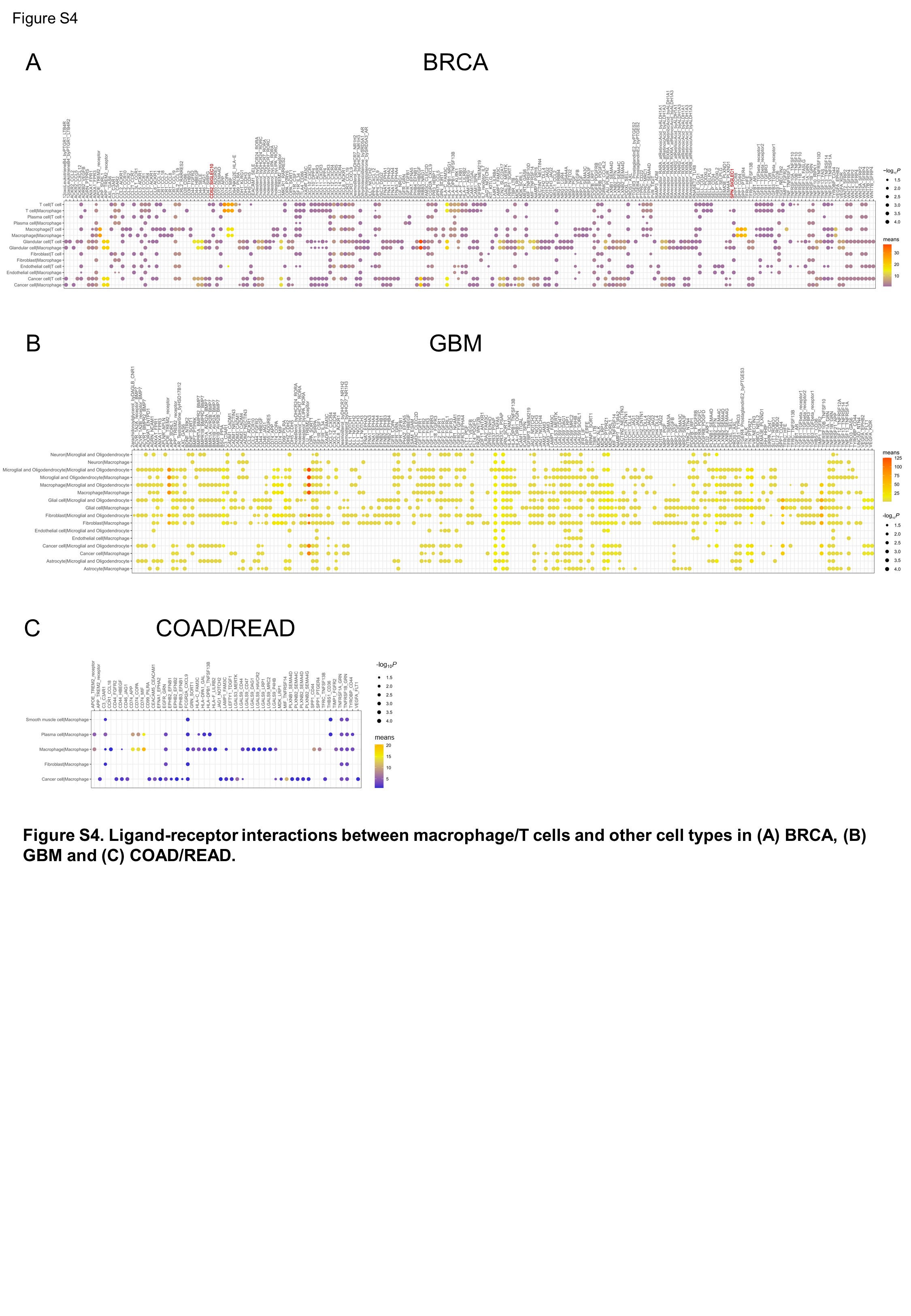

Supplement: Supplementary file 5 — Supporting Information [file CTM2-13-e1262-s004.jpg]
